# The MOVECLIM – AZORES project: Bryophytes from Terceira Island along an elevation gradient

**DOI:** 10.3897/BDJ.12.e131935

**Published:** 2024-09-05

**Authors:** Rosalina Gabriel, Leila Nunes Morgado, Débora Sofia Henriques, Márcia C. M. Coelho, Raquel Hernández-Hernández, Paulo A. V. Borges

**Affiliations:** 1 University of the Azores, cE3c- Centre for Ecology, Evolution and Environmental Changes/Azorean Biodiversity Group, CHANGE – Global Change and Sustainability Institute, School of Agricultural and Environmental Sciences, Rua Capitão João d´Ávila, Pico da Urze, 9700-042, Angra do Heroísmo, Azores, Portugal University of the Azores, cE3c- Centre for Ecology, Evolution and Environmental Changes/Azorean Biodiversity Group, CHANGE – Global Change and Sustainability Institute, School of Agricultural and Environmental Sciences, Rua Capitão João d´Ávila, Pico da Urze, 9700-042 Angra do Heroísmo, Azores Portugal; 2 IITAA - Instituto de Investigação e Tecnologias Agrárias e do Ambiente, Faculdade de Ciências Agrárias e do Ambiente, Universidade dos Açores, Angra do Heroísmo - Terceira Island / Azores, Portugal IITAA - Instituto de Investigação e Tecnologias Agrárias e do Ambiente, Faculdade de Ciências Agrárias e do Ambiente, Universidade dos Açores Angra do Heroísmo - Terceira Island / Azores Portugal; 3 Banco Genético Vegetal Autóctone, Empresa Municipal Cascais Ambiente, Lisboa, Portugal Banco Genético Vegetal Autóctone, Empresa Municipal Cascais Ambiente Lisboa Portugal; 4 Departamento de Botánica, Ecología y Fisiología Vegetal, Plant Conservation and Biogeography Group, Universidad de La Laguna, C/Astrofísico Francisco Sánchez, s/n. La Laguna, Islas Canarias, Spain Departamento de Botánica, Ecología y Fisiología Vegetal, Plant Conservation and Biogeography Group, Universidad de La Laguna, C/Astrofísico Francisco Sánchez, s/n. La Laguna Islas Canarias Spain; 5 IUCN SSC Atlantic Islands Invertebrate Specialist Group, Angra do Heroísmo, Azores, Portugal IUCN SSC Atlantic Islands Invertebrate Specialist Group Angra do Heroísmo, Azores Portugal; 6 IUCN SSC Monitoring Specialist Group, Angra do Heroísmo, Azores, Portugal IUCN SSC Monitoring Specialist Group Angra do Heroísmo, Azores Portugal

**Keywords:** Azores, AZU-Section Bryophytes, bryoflora, BRYOLAT, elevational gradient, liverworts, mosses, MOVECLIM-AZO, Natural Reserve, substrate, Terceira Island.

## Abstract

**Background:**

Systematic studies on the biodiversity of bryophytes along elevational gradients have been conductuted within the native vegetation of the Azores, using the MOVECLIM framework. The primary objective of this study was to inventory the bryophytes present within preserved areas of native vegetation in Terceira Island (Azores). From 25 to 28 September 2012, an inventory of the bryoflora was carried out along an elevational gradient, starting near Serreta lighthouse (38.76658 Latitude; -27.37539 Longitude; 40 m a.s.l.) and culminating on the top of Santa Bárbara Mountain (38.73064 Latitude; -27.32164 Longitude; 1000 m a.s.l.). The study followed the adapted MOVECLIM standardised protocol, as follows: i) six sites were selected along an elevational transect, each site spaced at 200 m elevation intervals; ii) within each site, two 10 m x 10 m plots were established in close proximity from each other (10-15 m); iii) within these plots, three 2 m x 2 m quadrats were randomly selected and sampled for bryophytes. The following substrates were surveyed in each quadrat: rock, soil, humus, organic matter, tree bark at three different heights and leaves/fronds. For each available and bryophyte-colonised substrate, three replicate microplots of 10 cm x 5 cm were collected, resulting in a maximum of 24 microplots per quadrat.

**New information:**

Nearly three-quarters of the maximum expected number of microplots (636 out of 864; eventID) were found across the six sites on Terceira Island, resulting in a total of 3677 records (occurrenceID). A high proportion of the specimens could be identified to the species rank (n = 3661; 99.6%), representing 38 families, 60 genera and 92 species, including 58 species of liverworts (Marchantiophyta) and 34 species of mosses (Bryophyta). The inventory included several endemic species: two liverwort species endemic to the Azores, five species endemic to Macaronesia (three mosses and two liverworts) and 11 European endemic species (three mosses and eight liverworts). The elevations with the highest species richness, the highest number of endemic species and the highest number of conservation concern species, spanned between 600 and 1000 m a.s.l. above sea level, coinciding with the best preserved forest vegetation. Overall, tree-dwelling and ground-dwelling substrates showed similar levels of bryophyte occupation (75% vs. 72%). However, the 636 events were unevenly distributed across substrates: leaves and rocks had the fewest replicates (n = 54; 50.0%), while humus and the lowest tree height had the highest values (n = 106; 98.1% and n = 98; 90.7%, respectively).

The study contributed to expanding knowledge about the diversity and distribution of the Azorean Bryoflora, both on a local and a regional scale.

## Introduction

Various factors, such as habitat loss and degradation, overexploitation, pollution, climate change and invasive species, have been affecting the integrity of ecosystems (Borges et al. 2020) and understanding how these factors influence biodiversity is especially critical in isolated environments like oceanic islands, that may serve as metaphors for planet Earth. Therefore, investigating the origin and diversification of island biota is an important objective of biogeography, evolutionary biology and conservation biology (Cowie and Holland 2006, Ricklefs 2010, Borregaard et al. 2016). Thus, it is important to characterise and monitor the performance of various taxonomic groups to adjust conservation strategies effectively and protect biodiversity now and in the future. The Azores, located in the North Atlantic Ocean, are one of the most isolated archipelagoes on Earth, lying more than 1200 km away from Europe and Africa and nearly 2000 km away from North America (Forjaz 2004). The nine islands, encompassing 2323 km^2^, are known for their unique terrestrial biota, with many species having evolved in isolation from continental areas. Presently, the archipelago is known to harbour around 6,112 terrestrial species, with 1,110 vascular plants species, 488 bryophytes and 788 lichens, while animals are the most represented kingdom with 3054 terrestrial species (Borges et al. 2010, ABP 2024). Of the 411 catalogued Azorean endemic species, 331 are animals, 73 are vascular plant species, seven are lichens and seven are bryophytes (Borges et al. 2010, Gabriel et al. 2011, Elias et al. 2022). The Azores stand out as one of the top three richest archipelagoes in bryophytes in the Atlantic (e.g. Costa and Sérgio (2023)), on par with Madeira (ca. 546 taxa, comprising six hornworts, 175 liverworts and 365 mosses; Martins et al. (2024)) and the Canary Islands (ca. 617 taxa, comprising six hornworts, 194 liverworts and 417 mosses; Hodgetts and Lockhart (2020)).

Bryophytes are a diverse group of ancient plants (Laenen et al. 2014), playing different important ecological roles, such as improving water availability (e.g. Coelho et al. (2023a), Coelho et al. (2023b)), promoting germination and seedling survival and reducing soil erosion (e.g. Glime (2024)), while serving as indicators of biodiversity and integrity of different ecosystem types (e.g. Frego (2007), Henriques et al. (2017a), Henriques et al. (2017b)). This group of plants is able to colonise different substrates, even impermeable ones such as rocks and leaves, as well as soil, humus, tree trunks and others (e.g. Sjögren (1997), Gabriel and Bates (2005)), exhibiting also complex and varied responses to elevation gradients (e.g. Henriques et al. (2016), Boch et al. (2019)).

Various studies have used elevation gradients to investigate biodiversity (see revision at Costa et al. (2023)). These gradients include a wide variety of habitats and a high number of bryophyte species within relatively small areas (Ah-Peng et al. 2012). Such a set-up allows researchers to infer the effects of varying temperature and humidity levels on bryophyte communities, that in turn affect the quality and composition of soil and other available substrates, anticipating change in environmental conditions and possible adaptation of biological communities (Ah-Peng et al. 2012, Coelho et al. 2023a).

Different elevation patterns have been observed in the distribution of bryophytes on islands. In Macaronesia, these tend to produce roughly mid-elevation peaks (e.g. Hernandez et al. (2017), Henriques et al. (2017a), Coelho et al. (2021), Martins et al. (2024)), coinciding with the highest humidity and forest cover, but other patterns may occur, including a pattern with two peaks found in La Reunion (Ah-Peng et al. 2012). These trends are influenced by a combination of climatic, ecological and historical factors (Gabriel et al. 2014). Understanding these patterns is essential for predicting the impacts of environmental changes and for developing effective conservation strategies to safeguard such hotspot areas (e.g. Stehn et al. (2010), Sim-Sim et al. (2014)).

Currently, the Azorean conservation policy is largely established by the Regional Legislative Decree no. 15/2012/A, of 2 April 2012 that created nine Natural Parks, one on each island of the archipelago, encompassing the classified areas of the Natura 2000 Network, as well as those covered by international conventions. With the creation of these Natural Parks, 124 areas are protected (19 Nature Reserves, 11 Natural Monuments, 48 Protected Areas for the Management of Habitats or Species, 16 Protected Landscape Areas and 30 Protected Areas for Resource Management), totalling 180,374 ha, of which 56,219 ha are land and 124,155 ha are marine areas (Direção Regional do Ambiente e Ação Climática 2020). The protected areas of the Azores include the most important terrestrial habitats of the archipelago, with special emphasis on middle and high elevation native forests. Nevertheless, even the legally protected areas may be vulnerable to anthropogenic threats, such as invasive species and global warming (Borges et al. 2020), threats that may endanger several conservation concern species found in these natural habitats (Dias et al. 2010).

In fact, oceanic volcanic islands, such as the Azores Archipelago, are particularly interesting study locations. Since 2012, substantial efforts have been dedicated to updating the information on bryophytes in the Azores, particularly through the MOVECLIM project 'Montane Vegetation as Listening Posts for Climate Change' (Gabriel et al. 2014, Borges et al. 2018, Coelho et al. 2021). This project aimed to characterise the highly diverse, but poorly known bryophyte flora across various archipelagoes (including the Azores, the Canary Islands and the Mascarene Islands) using standardised sampling on all available substrates on elevational gradients. In the Azores, seven islands were surveyed using an adapted version of the MOVECLIM protocol (Gabriel et al. 2014, Borges et al. 2018); specifically, Pico and Terceira Islands were surveyed in 2012, Flores and São Miguel Islands in 2013, São Jorge and Faial Islands in 2014 and Santa Maria Island in 2019.

A list of publications mentioning bryophytes collected in Terceira Island may be seen in Suppl. material 1. It comprises 162 references, published from 1844 to 2023, including mostly journal articles (112), but also other types of references, such as books and book chapters, theses and dissertations, scientific reports, Herbaria records and expert's documents. Rosalina Gabriel stands out with the highest number (32) of publications (author and co-author) on the inventory, distribution and ecology of Terceira Island's bryoflora. Authors such as Cecília Sérgio, Erik Sjögren and Eduardo Dias have contributed effectively to the knowledge of bryophytes on this Island. It is also important to mention Moritz A. Seubert, Jules Cardot and William Trelease, who began collecting and publishing records of bryophytes on Terceira Island in the 19^th^ century.

The knowledge of bryophyte diversity patterns along elevational gradients is useful for conservation management, especially in predicting how bryophyte communities might shift in response to environmental changes and in identifying key areas for protection on Azores Archipelago.

## General description

### Purpose

The main goal of this work is to inventory the bryoflora present in the Natural Park of Terceira using an adaptation of the MOVECLIM stratified protocol (Gabriel et al. 2014, Borges et al. 2018). Sampling followed the protocol across an elevation gradient from 40 m to 1000 m a.s.l. and included various substrates (rupicolous, terricolous, humicolous, lignicolous, epiphytic - at three heights from the soil and epiphyllous). This effort aims to expand the knowledge of bryophyte species (mosses, liverworts, hornworts) known on Terceira Island and the Azores and provide a baseline for future research on diversity.

## Project description

### Title

Inventory of bryoflora present in different altitudinal gradients and substrates of Terceira Island (Azores)

### Personnel

Conceptualisation

The project was conceived by the MOVECLIM Team, led by Claudine Ah-Peng and is currently being led by Rosalina Gabriel in the Azores. The MOVECLIM monitoring protocol, which roughly follows the BRYOLAT methodology, was conceived by Ah-Peng et al. (2012) and adapted for the Azores by RG and colleagues (Gabriel et al. 2014, Borges et al. 2018).

Fieldwork

Site selection and experimental setting: RG.

Permits: the Azores Government, through the Environment Department, gave the necessary authorisations to work within the Natural Park of Terceira.

Sample collection: The bryoflora inventory of Terceira Island was conducted from 25-28 September 2012, under the responsibility of RG, with the participation of DH, MC, RH and Fernando Pereira.

Lab work

Taxonomic work: All taxa were identified by DH, supervised by RG, in 2013-2014; some samples were confirmed by Cecília Sérgio in 2015. In 2023-2024, specimens of the genus *Frullania* were reviewed by LNM, with confirmation by Manuela Sim-Sim for challenging samples.

Management

Voucher specimen management: DH, MC and RG.

Database management: RG.

Darwin Core databases: LNM, PB and RG.

### Study area description

The archipelago of the Azores is an autonomous region of Portugal, located in the north Atlantic Ocean (36°55’–39°43’ N and 25°00’–31°15’ W), about 1500 km from the western coast of mainland Europe and approximately 3900 km from the North America coasts, lying at a triple junction amongst three lithospheric plates: the Eurasian, American and African plates (Anonymous 1974, Forjaz 2004). The nine islands, all of volcanic origin, are organised in three island groups: Eastern (Santa Maria and São Miguel Islands), Central (Terceira, Graciosa, São Jorge, Pico and Faial Islands) and Western (Flores and Corvo Islands).

**Figure 1.**
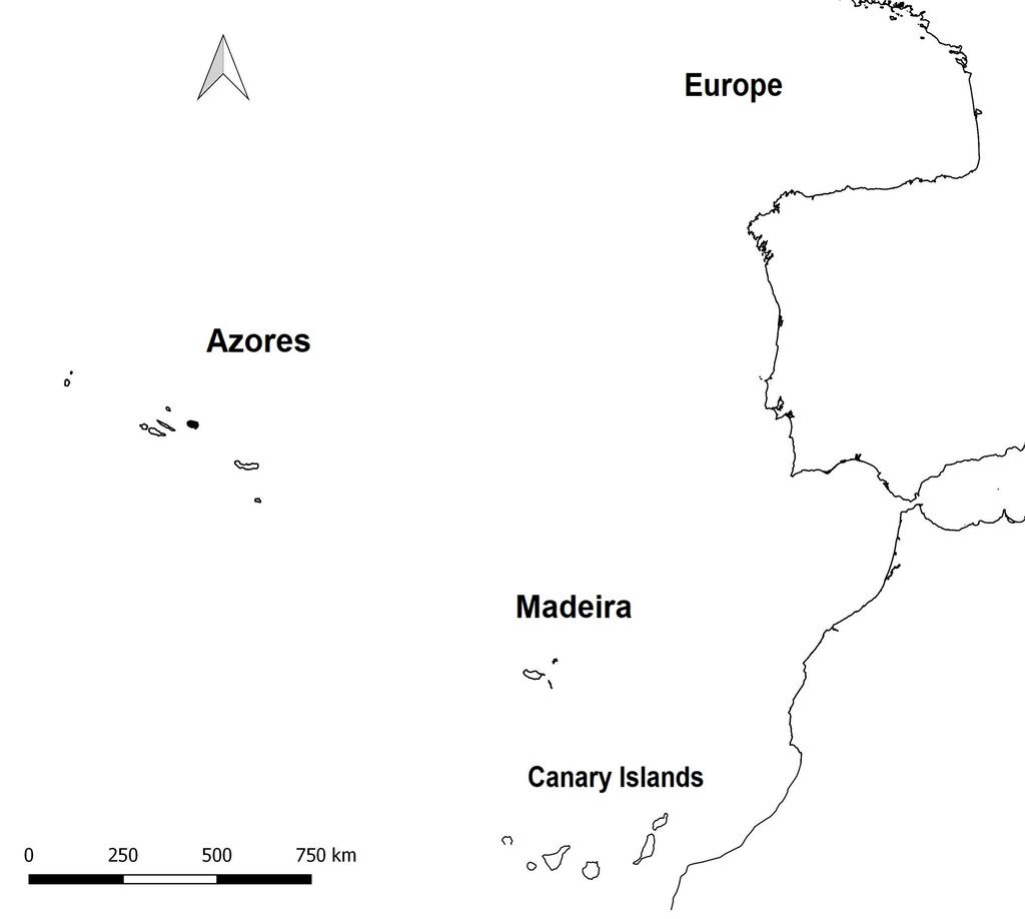
Location of the Azores Archipelago in relation to the Iberian Peninsula, Madeira Archipelago and the Canary Islands.

The Azores are part of the Macaronesian Region, which also includes Madeira, the Canary Islands and Cape Verde (Fig. 1) (Forjaz 2004).

The Azores Islands have a total area of 2,323 km², with maximum elevations ranging from 405 m (Graciosa Island) and 2,351 m (Pico Island), while the elevations on the other islands average around 1,000 m (Forjaz 2004). The islands experience hyper-humid, oceanic, supra-oceanic and alpine climates, the latter restricted to Pico mountain (Rivas-Martínez 2008), with a mean annual temperature of 17ºC at sea level. Annual rainfall ranges between 1,500 and 3,000 mm and the islands maintain high relative humidity (Azevedo 2001).

Terceira Island, in the Central Group, is situated at Latitude 38°43′0″ N and Longitude 27°12′0″ W. The Island spans approximately 29 km in length and 18 km in width, with a perimeter of 90 km. It covers an area of approximately 402.2 km^2^ and its highest point, at 1,021 m a.s.l., is located in the Santa Bárbara Mountain range on the west side (Forjaz 2004). The Island is characterised by high relative humidity and mild temperatures with low fluctuations throughout the year (Azevedo 2001); in the Santa Bárbara Mountain, the average annual rainfall exceeds 3,400 mm and the mean temperature is 9°C (Azevedo et al. 2004).

Most of the natural vegetation in the Azores has been transformed by human activity, especially affecting Laurel forests, including habitat destruction (e.g. Borges et al. (2009)) and decharacterisation, especially due to the increase in exotic species (e.g. Sousa et al. (2024)). The best preserved vegetation is found above 600 m a.s.l., including *Juniperus-Ilex* forests and *Juniperus* woodlands (Elias et al. 2016). On Terceira Island, most of the land is occupied by pastures, with only about a tenth covered by native forests (Borges et al. 2009) and about a fifth (22.07%) legally protected (Direção Regional do Ambiente e Ação Climática 2020). The natural vegetation is concentrated in the central areas at higher elevations, corresponding to *Juniperus-Ilex* montane forest and *Calluna*-*Juniperus* altimontane scrubland formations (Elias et al. 2016), but a few remnants of native vegetation may still be found from the coast to 400 m a.s.l., especially on the western slope.

Bryophytes are an important part of the native vegetation of Terceira Island. The distribution of species is related to environmental variables such as water availability and temperature, the presence of different substrates and the vascular plant community (e.g. Gabriel and Bates (2005)). For instance, a recent study in the Terceira Island Natural Park, the 'Matela Protected Area for the Management of Habitats or Species', accounted for a total of 75 bryophyte taxa (44 mosses and 32 liverworts) within an area less than 27 ha (Sousa et al. 2024).

**Figure 2.**
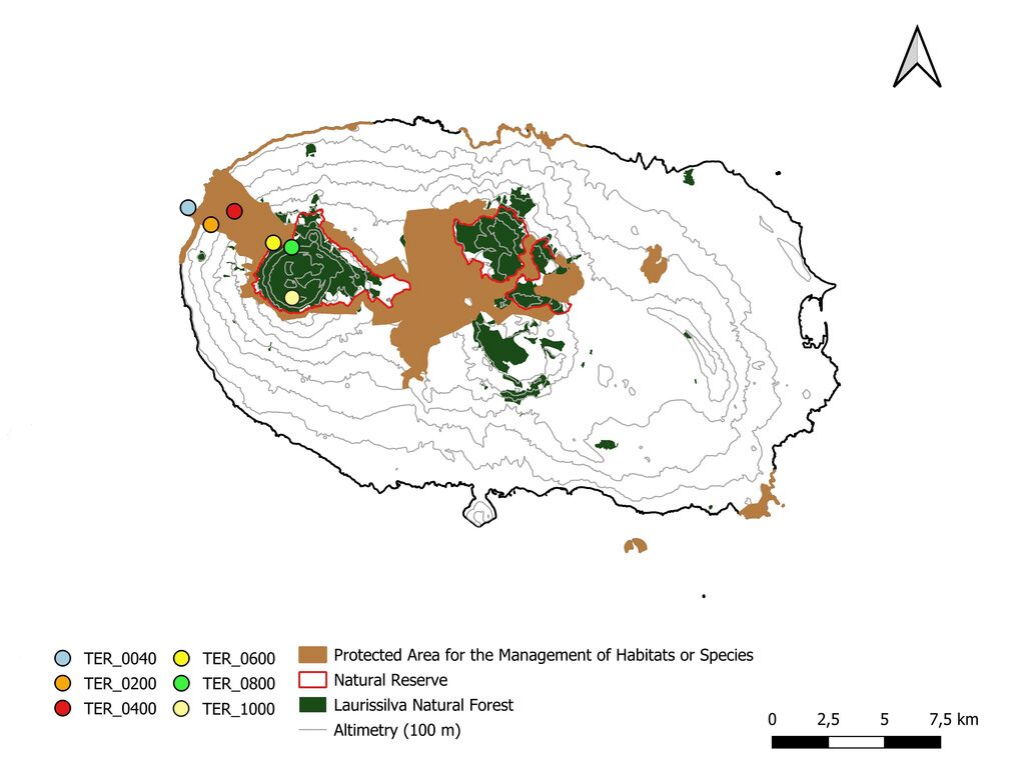
**Figure 2.** Map of Terceira Island showing the sampling points and delimitation of the Protected Area for the Management of Habitats or Species and the Natural Reserve, including the Laurissilva Natural Forest area.

The sampling location and coordinates are listed in Table 1 and Fig. 2.

### Design description

The sampling was performed in 2012, during 25-28 September, along a longitudinal elevational transect in the Natural Park of Terceira (Regional Legislative Decree no. 11/2011/A, of 20 April 2011) in the western part of the Island, including the preserved areas of native vegetation (Nature Reserve and Protected Area for the Management of Habitats or Species). The samples were collected in different elevation levels, from 40 to 1000 m a.s.l. (Fig. 2), on all available substrates (rupicolous, terricolous, humicolous, lignicolous, epiphytic, epiphyllous).

### Funding

This study was financed by ERANET BIOME MOVECLIM – ‘Montane vegetation as listening posts for climate change’ of the regional government of the Azores, grant number M2.1.2/F/04/2011/NET. MC was funded by the FUNDO REGIONAL PARA A CIÊNCIA E TECNOLOGIA (FRCT) of the Regional Government of the Azores, grant number M3.1.2/F/007/2012. RG and PAVB are currently funded by FCT-UIDB/00329/2020-2024, DOI 10.54499/UIDB/00329/2020 (Thematic Line 1–integrated ecological assessment of environmental change on biodiversity) and Azores DRCT Pluriannual Funding (M1.1.A/FUNC.UI&D/010/2021-2024). The AZORESBIOPORTAL funded data curation in last six years through FEDER at 85% and by regional funds at 15%, via the Azores 2020 Operational Programme, through the “PORBIOTA-AZORES BIOPORTAL” project (ACORES-01-0145-FEDER-000072) (2019-2022). LNM is funded by Centre for Ecology, Evolution and Environmental Changes (cE3c), with base funding ref. UIDB/00329/2020-2024 - Fundação para a Ciência e Tecnologia, I.P.(FCT).

## Sampling methods

### Study extent

This study was conducted in the Protected Area for the Management of Habitats or Species and the Santa Bárbara Natural Reserve, the latter part of the Special Conservation Zone (ZEC) of the Natura 2000 Network. Both areas are included in the Natural Park of Terceira Island, which is part of the Regional Network of Protected Areas of the Autonomous Region of the Azores (Direção Regional do Ambiente e Ação Climática 2020). The study area features a diverse range of vegetation types and is characterised by a high diversity of native and endemic species, protected habitats and ecosystems. Notable vegetation includes pioneer forests of *Juniperusbrevifolia* (Seub.) Antoine and *Laurusazorica* (Seub.) Franco, with a high cover of bryophytes, including *Sphagnum* spp., amongst other botanical species. The Santa Bárbara Mountain range, consisting of a double volcanic caldera, hosts many endemic and rare vascular plant species, including 18 Azorean endemics, five Macaronesian endemics, 10 species protected by the Bern Convention and seven species protected under the Habitat and Species Directive (Dias et al. 2004).

### Sampling description

The field study was conducted using an adapted MOVECLIM methodology, modified, based on knowledge of the vascular flora and topography of Terceira Island (Gabriel et al. 2014). Sampling followed an elevation gradient with 200 m intervals, ranging from the coast (at 40 m a.s.l.) to the summit of Santa Bárbara Mountain (at 1000 m a.s.l.). At each site, two 10 m x 10 m plots were established and, from each plot, three 2 m x 2 m quadrats were randomly selected for surveying. Bryophytes were sampled within a total of three microplots (5 cm x 10 cm) per substrate type (rupicolous, terricolous, humicolous, lignicolous, epiphytic — at three heights, and epiphyllous) across the altitudinal gradient.

In the laboratory, the microplots were analysed, bryophytes were identified to the species level and their abundance and sociability values were estimated.

### Quality control

FIELD: Plots were placed within homogeneous areas of the most representative native vegetation found at each sampled elevation. Sampling was made by experienced bryologists, who ensured the samples were properly collected, while avoiding the excessive removal of material.

STORAGE: After collection of the the microplot samples for paper bags, these were left open and separated in a darkened room until complete dehydration. After identification, every sample was transferred to herbarium envelopes properly identified. All these envelopes were stored on the Herbarium of the University of the Azores (AZU), Section Bryophytes, under the name 'MOVECLIM – AZORES project: Bryophytes from Terceira Island (2012)'.

TAXONOMY: All efforts were made to achieve an accurate identification of the specimens: (i) the most updated keys and floras were used by/under the supervision of experienced bryologists; (ii) challenging samples were sent to specialists for confirmation/identification; (iii) identification of extremely small or etiolated specimens was not pursued to the species level. Nomenclature followed Hodgetts et al. (2020).

Mosses were identified using the floras of Smith (2004) and Casas et al. (2006), whereas liverworts were identified using the floras written by Paton (1999) and Casas et al. (2009) and the taxonomic key of Schumacker and Vána (2005). Visual guides (e.g. Atherton et al. (2010), Lüth (2019)) were also consulted, as well as the BBS Field Guide *online* pages, the *Bildatlas der Moose Deutschlands* for morphological and ecological data. Nomenclature follows Gabriel et al. (2010) and adaptations available on the Borges et al. (2024).

Species identification was mainly performed by Débora S.G. Henriques, under the supervision of Rosalina Gabriel. In 2023-2024, all the *Frullania* specimens were reviewed by Leila Nunes Morgado under the supervision of Rosalina Gabriel. Cecília Sérgio and Manuela Sim-Sim have reviewed some challenging material.

### Step description

**PART 1. Obtaining samples**

1. **Conceptualisation**: Develop a research design to enhance the understanding of the bryoflora in native habitats of Terceira Island, Azores, Portugal.

2. **Site Selection**: Choose six sites along an elevational transect on Terceira Island, using a 200 m step and focusing on areas with the highest cover of native vascular plant species.

3. **Plot Selection**: At each site, select two study plots (10 m x 10 m), spaced 10-15 m apart within a homogeneous vegetation area; describe plots using different indicators, such as the general cover of vascular plant species and bryophytes, height and dbh of the 10 tallest trees, aspect, inclination etc.

4. **Quadrat Selection**: In each plot, randomly select three quadrats (2 m x 2 m) for detailed inspection.

5. **Substrate Examination**: In each selected quadrat, examine all available substrates, including rock, soil, humus, organic matter, bark and leaves/fronds. Collect three samples (replicates; microplots) (10 cm x 5 cm) from each substrate.

6. **Drying**: Dry the collected material in an airy, darkened, room to preserve the specimen.

7. **Identification**: Using the available keys and floras, identify the species present in each microplot.

8. **Registration**: Register the data on a database.

9. **Herbarium Storage**: Store the identified samples in the Herbarium of the University of the Azores – Section Bryophytes (AZU-B).

**PART 2. Data Preparation and Sharing**:

Data files were prepared to share information regarding Terceira Island bryophytes with GBIF and other platforms, using Darwin Core Archive (DwC-A), which is a standardised format for sharing biodiversity data as a set of one or more data tables.

1. **Event Table Preparation**: Prepare a core data table (events), containing the 636 records, corresponding to each 10 cm x 5 cm microplot.

2. **Occurrence Data Table Preparation**: Prepare a data table extension (occurrence) with 3677 records, detailing the inventory of all bryophytes found in the microplots.

3. **Revision**: Ensure all data is formatted according to the Darwin Core Archive (DwC-A) standards for sharing biodiversity data.

4. **Sharing**: Share the information regarding Terceira Island bryophytes with GBIF and other platforms using the standardised DwC-A format.

## Geographic coverage

### Description

The study was carried out in Terceira Island (Azores Archipelago, Portugal). The six sampling sites were distributed in the Natural Park of Terceira, Municipality of Angra do Heroísmo.

### Coordinates

38.73047 and 38.76658 Latitude; -27.32164 and -27.37539 Longitude.

## Taxonomic coverage

### Description

Bryophytes, including specimens from Phylum Bryophyta (mosses) and Phylum Marchantiophyta (liverworts). No representatives from Phylum Anthocerotophyta were collected during this survey.

## Temporal coverage

### Notes

The sampling was performed during 25-28 September 2012.

## Collection data

### Collection name

MOVECLIM-AZO-TER_2012_Bryophytes from Terceira Island

### Collection identifier

76349556-a70a-4ecc-88a7-cd085b6c875d

### Parent collection identifier

AZU_Section Bryophytes

### Specimen preservation method

Herbarium preservation

### Curatorial unit

Herbarium packet

## Usage licence

### Usage licence

Creative Commons Public Domain Waiver (CC-Zero)

### IP rights notes

Additional information on this study may also be requested from the corresponding author.

## Data resources

### Data package title

The MOVECLIM – AZORES project: Bryophytes from Terceira Island (2012).

### Resource link

https://doi.org/10.15468/ud4xhq

### Alternative identifiers

https://www.gbif.org/dataset/d6994db0-3824-425d-8cb0-360be1bbc46a

### Number of data sets

2

### Data set 1.

#### Data set name

Event table

#### Data format

Darwin Core Archive

#### Character set

text unicode

#### Download URL

http://ipt.gbif.pt/ipt/resource?r=moveclim_az_terceiraisland_2012&v=1.4

#### Data format version

1.4

#### Description

The dataset was published in the Global Biodiversity Information Facility platform, GBIF (Gabriel et al. 2024). The following data table includes all the records for which a taxonomic identification of the species was possible. The dataset submitted to GBIF is structured as a sample event dataset that has been published as a Darwin Core Archive (DwCA), which is a standardised format for sharing biodiversity data as a set of one or more data tables. The core data file contains 636 records (eventID). This GBIF IPT (Integrated Publishing Toolkit, Version 2.5.6) archives the data and, thus, serves as the data repository. The data and resource metadata are available for download in the Portuguese GBIF Portal IPT (Gabriel et al. 2024).

**Data set 1.** 

Column label	Column description
eventID	Identifier of the events, unique for the dataset.
type	Type of the record, as defined by the Dublin Core Standard.
datasetName	Name of the dataset that in current projects is "MOVECLIM-AZO_2012_Bryophytes from Terceira Island".
dynamicProperties	Orientation and display of the samples in each plot (Aspect and inclination of the surface). Aspect is the orientation of slope, measured clockwise in degrees from 0 to 360, where 0 is north-facing, 90 is east-facing, 180 is south-facing and 270 is west-facing.
samplingProtocol	The sampling protocol used to capture the species. Two plots of 10 m × 10 m (P1, P2) were established at natural vegetation sites every 200 m in elevation, ranging from 40 m at Serreta lighthouse to 1000 m at the summit of Santa Bárbara Mountain. Each plot was divided into 25 quadrats of 2 m × 2 m, from which three (Q1, Q2, Q3) were randomly selected for bryophyte species collection. Within each quadrat, bryophytes were sampled in small units (microplots of 10 cm × 5 cm), with three replicates per surveyed substrate whenever possible. The substrates included rock (RU), soil (TE), humus (HU), dead wood (LI) and bark at three heights from the tree base (TA: 1-50 cm; TB: 51-100 cm; TC: 101-200 cm), as well as leaves/fronds (LF).
eventDate	The date-time or interval during which an Event occurred. For occurrences, this is the date-time when the event was recorded.
year	Year the sample was collected (2012).
habitat	The habitat for an Event.
continent	The name of the continent in which the Location occurs (Europe).
islandGroup	The name of the island group in which the Location occurs (Azores).
island	The name of the island on or near which the Location occurs (Terceira Island).
country	The name of the country or major administrative unit in which the Location occurs (Portugal).
countryCode	The standard code for the country in which the Location occurs (PT).
municipality	The full, unabbreviated name of the next smaller administrative region than county (city, municipality etc.) in which the Location occurs.
locality	The specific description of the place.
verbatimElevation	The original description of the elevation (altitude above sea level in metres) of the Location; this information was obtained with a GARMIN GPS with barometric correction.
verbatimCoordinates	Original coordinates recorded.
decimalLatitude	Approximate centre point decimal latitude of the field site in GPS coordinates.
decimalLongitude	Approximate centre point decimal longitude of the field site in GPS coordinates.
geodeticDatum	Standard Global Positioning System coordinate reference for the location of the sample collection points.
coordinateUncertaintyInMetres	Uncertain value of coordinate metrics.
coordinatePrecision	Value in decimal degrees to a precision of five decimal places.
georeferenceSources	Navigation system used to record the location of sample collections.

### Data set 2.

#### Data set name

Occurrence Table

#### Data format

Darwin Core Archive

#### Character set

text unicode

#### Download URL

http://ipt.gbif.pt/ipt/resource?r=moveclim_az_terceiraisland_2012&v=1.4

#### Data format version

1.4

#### Description

The dataset was published in the Global Biodiversity Information Facility platform, GBIF (Gabriel et al. 2024). The following data table includes all the records for which a taxonomic identification of the species was possible. The dataset submitted to GBIF is structured as an occurrence table that has been published as a Darwin Core Archive (DwCA), which is a standardised format for sharing biodiversity data as a set of one or more data tables. The core data file contains 3677 records (occurrenceID). This GBIF IPT (Integrated Publishing Toolkit, Version 2.5.6) archives the data and, thus, serves as the data repository. The data and resource metadata are available for download in the Portuguese GBIF Portal IPT (Gabriel et al. 2024).

**Data set 2.** 

Column label	Column description
eventID	Identifier of the events, unique for the dataset.
licence	Reference to the licence under which the record is published.
institutionID	The identity of the institution publishing the data.
institutionCode	The code of the institution publishing the data.
collectionID	Identifier of the collection, unique for each specimens are conserved.
collectionCode	The code of the collection where the specimens are conserved.
datasetName	Project reference.
type	Characteristics of the object of study.
basisOfRecord	The nature of the data record.
dynamicProperties	A list of additional measurements, facts, characteristics or assertions about the record, including IUCN categories (Endangered, Vulnerable, Near Threatened, Least Concern, Not Evaluated) and colonisation status of taxa following the standard notation used for bryophytes (Azorean endemic, Macaronesian endemic, Ibero-Macaronesian endemic, European endemic, non-endemic).
occurrenceID	Identifier of the record, coded as a global unique identifier.
recordNumber	An identifier given to the Occurrence at the time it was recorded.
recordedBy	A list (concatenated and separated) of names of people, groups or organisations responsible for recording the original Occurrence.
identifiedBy	A list (concatenated and separated) of names of people, who made the identification.
dateIdentified	date of species identification.
disposition	The current state of a specimen with respect to the collection identified in collectionCode or collectionID.
taxonRank	Lowest taxonomic rank of the record.
kingdom	Kingdom name.
phylum	Phylum name.
class	Class name.
order	Order name.
family	Family name.
genus	Genus name.
specificEpithet	Specific epithet.
infraspecificEpithet	Infraspecific epithet at subspecies level.
scientificNameAuthorship	The authorship information for the scientificName formatted according to the conventions of the applicable nomenclaturalCode.
scientificName	Complete scientific name including author.
organismQuantity	A number or enumeration value for the quantity of organisms (i, solitary specimen - one or few individuals; p, occasional and less than 5% cover; 1, less than 5% cover of total area; 2, 5%-25% of total area; 3, 25%-50% of total area; 4, 50%-75% of total area; 5, 75%-100% of total area).
organismQuantityType	Braun-Blanquet Scale.
establishmentMeans	The process of establishment of the species in the location, using a controlled vocabulary: 'native non-endemic', 'introduced', 'endemic'.
occurrenceRemarks	Remarks on the occurrence substrate from where the specimens were captured.

## Additional information

**Figure 3.**
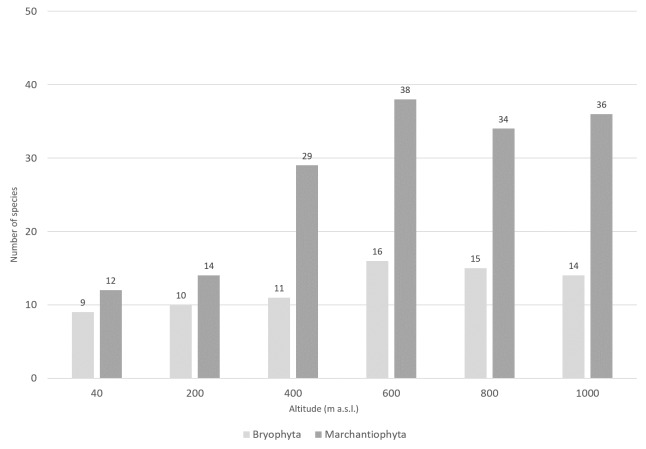
Number of bryophyte species (mosses: Bryophyta and liverworts: Marchantiophyta) along the elevational gradient of Terceira Island studied using the MOVECLIM Protocol in 2012.

**Figure 4.**
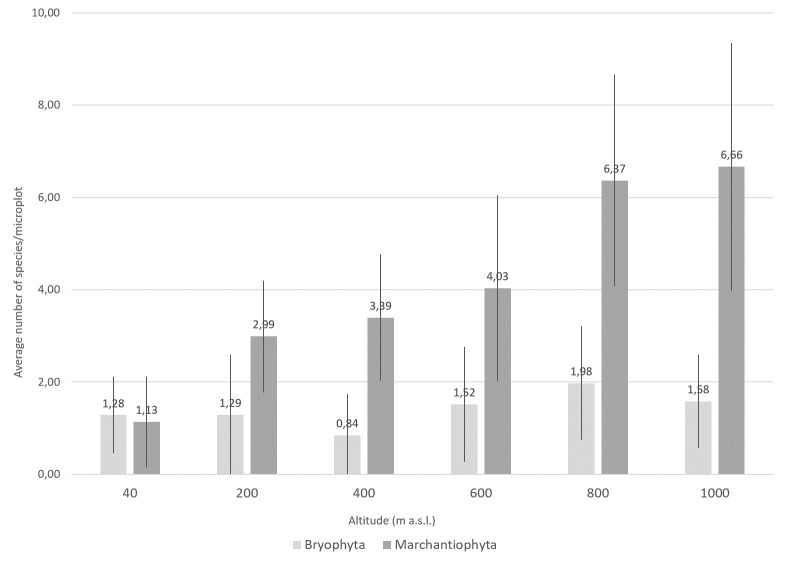
Average number of bryophyte species (mosses: Bryophyta and liverworts: Marchantiophyta) along the elevational gradient of Terceira Island studied using the MOVECLIM Protocol in 2012.

The 636 events yielded a grand total of 3677 specimens, with the majority (n = 3661; 99.6%) being successfully identified down to the species/subspecies level. Phylum Bryophyta (Table 2a) is represented by 34 species, including 24 genus, 18 families, seven orders and three classes (Bryopsida, Polytrichopsida and Sphagnopsida). Phylum Marchantiophyta (Table 2b) is represented by 58 species, including 36 genus, 20 families, 11 orders and two classes (Jungermanniopsida and Marchantiopsida) (see also Occurrence Table at Gabriel et al. (2024)). Liverworts are thus a more diverse group than mosses within the studied system, both in richness of species (Fig. 3) and number of species present per microplot (Fig. 4). The overall liverwort-to-moss ratio is 1.71.

**Figure 5.**
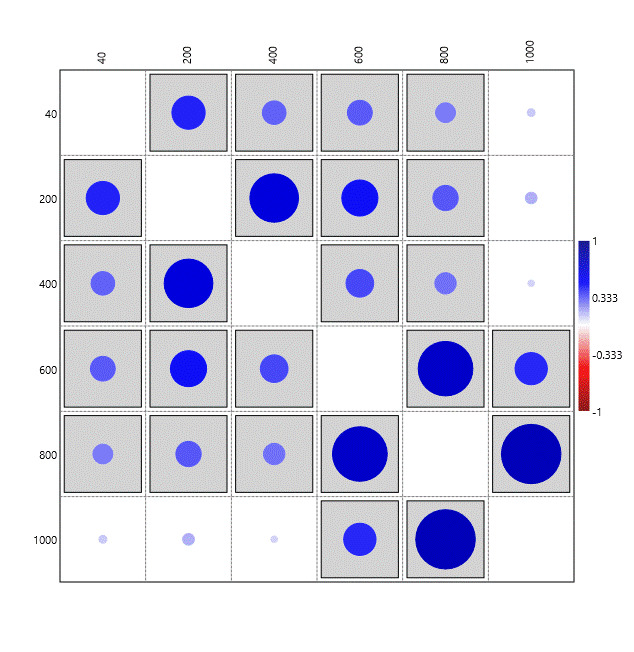
Correlation between relative abundances of bryophytes on different elevations (m a.s.l.). The analysis was conducted using the "correlation" function of PAST (PAleontological STatistics; Hammer et al. (2001)). Statistically significant correlations (p < 0.05) are indicated by boxed squares. The intensity of colour and size of circles represent the Pearson correlation coefficient (R), with R = 1 (maximum positive correlation) shown by the largest and darkest circles.

A pairwise correlation analysis using the function "correlation" of PAST (PAleontological STatistics; Hammer et al. (2001)), revealed that bryophyte composition tends to correlate, in higher or lower degree, along the elevation gradient (Fig. 5), with the highest correlation values occurring between neighbouring elevation levels. However, the island's summit, at 1000 m a.s.l., shows a statistically significant correlation only with the 600 m and 800 m elevation sites.

Considering the colonisation status, all the taxa can be considered native, but two liverworts are Azoren endemics, *Bazzaniaazorica* H.Buch & Perss. and Leptoscyphusporphyriussubsp.azoricus (H.Buch & Perss.) Vanderp. & Heirichs, five species are Macaronesian endemics and 11 are European endemics (see Table 2).

Some species are considered conservation concern using the IUCN Red Listing criteria (Hodgetts 2019): one moss (*Daltonialindigiana* Hampe) and five liverworts (*Bazzaniaazorica* H.Buch & Perss., *Cololejeuneasintenisii* (Steph.) Pócs, *Herbertusazoricus* (Steph.) P.W.Richards, Leptoscyphusporphyriussubsp.azoricus (H.Buch & Perss.) Vanderp. & Heinrichs and *Telaraneaazorica* (H.Buch & Perss.) Pócs) are considered endangered, five other species are considered vulnerable and eight species are considered near threatened (see Table 2). Nevertheless, *Cheilolejeuneacedercreutzii* (H.Buch & Perss.) Grolle, a liverwort species considered a TOP 100 priority species for conservation (Cardoso et al. 2008) was not found in 2012, although it was referred to Santa Bárbara Mountain in 1998 (Gabriel and Bates 2005).

**Figure 6.**
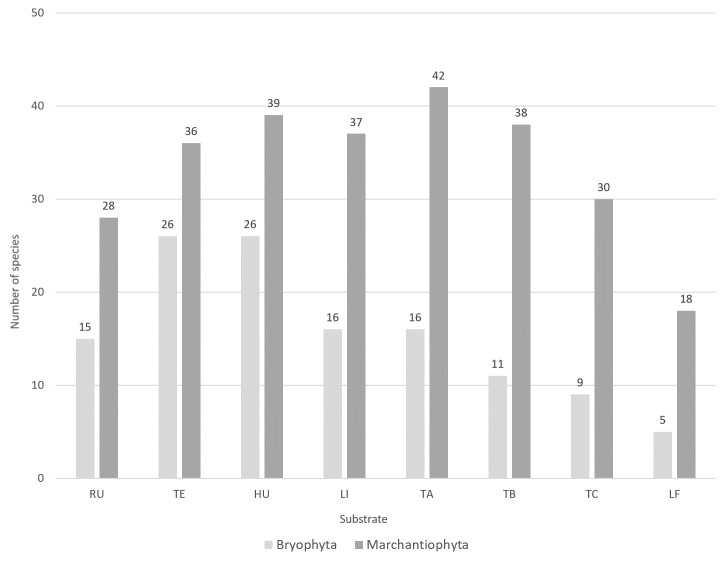
Number of bryophyte species (mosses: Bryophyta and liverworts: Marchantiophyta) across different substrates found along the elevational gradient of Terceira Island studied using the MOVECLIM Protocol in 2012 (RU, rupicolous; TE, terricolous, HU, humicolous; LI, lignicolous; TA, epiphytic from 1-50 cm from the soil; TB, epiphytic from 51-100 cm from the soil; TC, epiphytic from 101-200 cm from the soil; LF, epiphyllous).

**Figure 7.**
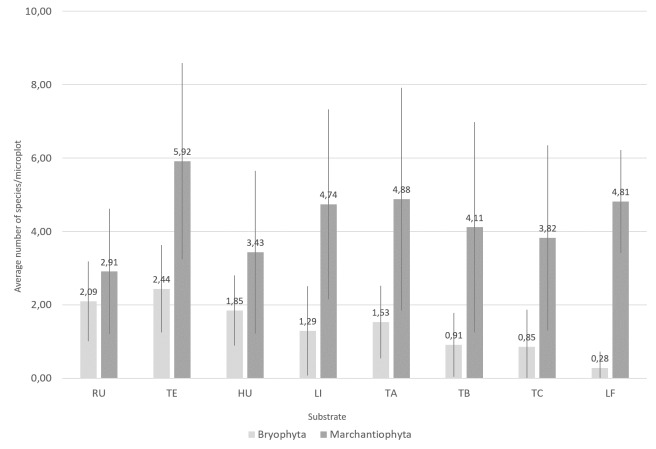
Average number of bryophyte species (mosses: Bryophyta and liverworts: Marchantiophyta) across different substrates found along the elevational gradient of Terceira Island studied using the MOVECLIM Protocol in 2012 (RU, rupicolous; TE, terricolous, HU, humicolous; LI, lignicolous; TA, epiphytic from 1-50 cm from the soil; TB, epiphytic from 51-100 cm from the soil; TC, epiphytic from 101-200 cm from the soil; LF, epiphyllous).

The highest species richness corresponds to sites with more complex vegetation (Henriques et al. 2017a, Henriques et al. 2017b). Notably, almost two-thirds of the liverworts (n = 38; 65.5%) and nearly half of the mosses (n = 16; 47.1%) were collected at 600 m a.s.l. (Fig. 3), with abundance peaking at 800 m altitude (Henriques et al. 2017a). Both mosses and liverworts exhibit comparable distribution patterns, but liverworts consistently show greater species richness and abundance than mosses across all elevation levels and substrates (Figs 6, 7), as was shown by Henriques et al. (2016). Amongst epiphytic substrates, the base of the trees (TA, 1-50 cm) is the richest for liverworts, harbouring 42 of the 58 liverwort species (Fig. 6). Conversely, humicolous and terricolous substrates are more species-rich for mosses, each supporting 26 of the 34 moss species (Fig. 6).

**Figure 8.**
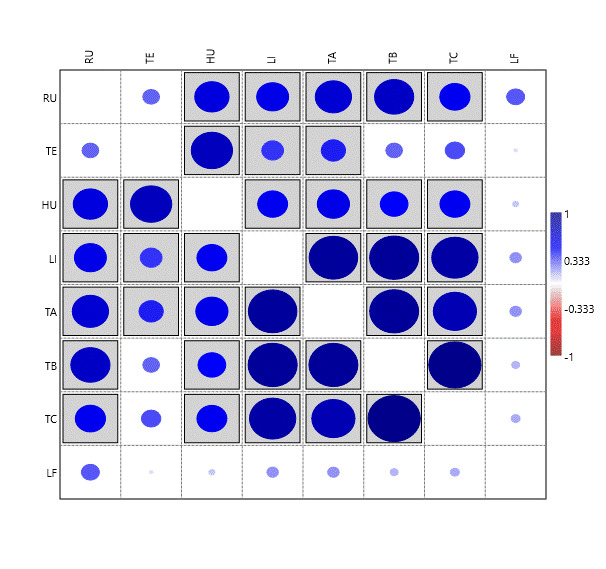
Correlation between relative abundances of bryophytes on different substrates (RU, rupicolous; TE, terricolous; HU, humicolous; LI, lignicolous; TA, epiphytic from 1-50 cm from the soil; TB, epiphytic from 51-100 cm from the soil; TC, epiphytic from 101-200 cm from the soil; LF, epiphyllous). The analysis was conducted using the "correlation" function of PAST (PAleontological STatistics; Hammer et al. (2001)). Statistically significant correlations (p < 0.05) are indicated by boxed squares. The intensity of colour and size of circles represent the Pearson correlation coefficient (R), with R = 1 (maximum positive correlation) shown by the largest and darkest circles.

Different bryophyte species were found amongst the eight different substrates. However a pairwise correlation analysis, using the function "correlation" of PAST (PAleontological STatistics) (Hammer et al. 2001), revealed that, except for the epiphylls (LF), which are never statistically correlated with the other communities, the remaining substrates show some degree of correlation (Fig. 8). Tree bark samples, either epiphytic or lignicolous, show the highest internal correlation. Interestingly, the samples collected in trees above 50 cm (TB and TC) show the lowest correlation values with terricolous species (TE).

### Conclusion

The MOVECLIM Protocol applied in 2012 to a transect on Terceira Island revealed a diverse ecosystem with indigenous bryophyte species thriving on various substrates, both permeable and impermeable, across ground level and within the vertical structure of the forest. The high liverwort-to-moss ratio (1.71) suggests prevalent wet and shaded conditions typical of cloud forests and these conditions harbour the presence of endemic and conservation concern species.

As anticipated (Gabriel and Bates 2005, Henriques et al. 2017a, Henriques et al. 2017b), there is a clear positive correlation between vegetation complexity and bryophyte species richness: sites with more intricate vegetation structures support greater numbers of bryophyte species, especially liverworts. Consequently, the highest richness values were observed from 600-1000 m a.s.l., where more than 85% of the surveyed microplots were effectively occupied by bryophytes.

In terms of substrate preference, liverworts exhibit highest species richness on epiphytic substrates, particularly at the base of trees. Conversely, mosses are more species-rich on humicolous and terricolous substrates. This disparity underscores distinct ecological differences between liverworts and mosses and may imply different conservation strategies for the two taxonomic groups.

## Supplementary Material

AA290276-3B69-5321-9720-FA2C3C83995C 10.3897/BDJ.12.e131935.suppl1 Supplementary material 1 List of publications mentioning bryophytes in Terceira Island (Azores) from 1844 to 2023 Data type Table Brief description List of references mentioning the distribution of bryophytes (mosses, liverworts and hornworts) in Terceira Island (Azores, Portugal), from 1844 to 2023. Each row includes information on the year of publication, author(s), full reference and type of publication. File: oo_1090459.txt https://binary.pensoft.net/file/1090459 Rosalina Gabriel

## Figures and Tables

**Table 1.** Information regarding the bryophyte sampling sites of the MOVECLIM project on Terceira Island, Azores, including plot code, locality name, elevation (in metres a.s.l.) and coordinates (in decimal degrees).

Plot Code	Locality	Elevation (m a.s.l.)	Latitude	Longitude
TER_0040_P1	Serreta, Lighthouse	31	38.76658	-27.37539
TER_0040_P2		38	38.76653	-27.37514
TER_0200_P1	Canada das Covas, Serreta	243	38.75981	-27.36364
TER_0200_P2		229	38.76005	-27.36392
TER_0400_P1	Carneiro, Peak	387	38.76522	-27.35164
TER_0400_P2		381	38.76501	-27.35162
TER_0600_P1	Lagoinha, Peak	675	38.75258	-27.33164
TER_0600_P2		675	38.75253	-27.33136
TER_0800_P1	Lagoa do Pinheiro, Trail	824	38.75083	-27.32222
TER_0800_P2		827	38.75086	-27.32203
TER_1000_P1	Santa Bárbara, Mountain	988	38.73047	-27.32205
TER_1000_P2		990	38.73064	-27.32164

**Table 2.** List of sampled species and subspecies in each of the colonisation status categories (Azorean endemic; Macaronesian endemic; Ibero-Macaronesian endemic; European endemic; Native) and IUCN Status (Endangered; Vulnerable; Near Threatened; Least Concern).

**Colonization status**	**Scientific Name**	**IUCN Status**
		
**a) Phylum: Bryophyta**		
Macaronesian endemic	*Andoaberthelotiana* (Mont.) Ochyra	Vulnerable
	*Isotheciumprolixum* (Mitt.) M.Stech, Sim-Sim, Tangney & D.Quandt	Vulnerable
	*Tetrastichiumvirens* (Cardot) S.P.Churchill	Near Threatened
European endemic	*Hypnumuncinulatum* Jur.	Least Concern
	*Pseudotaxiphyllumlaetevirens* (Dixon & Luisier ex F.Koppe & Düll) Hedenäs	Near Threatened
	*Tetrastichiumfontanum* (Mitt.) Cardot	Vulnerable
Native	*Brachytheciastrumvelutinum* (Hedw.) Ignatov & Huttunen	Least Concern
	*Brachytheciumrutabulum* (Hedw.) Schimp.	Least Concern
	*Campylopusbrevipilus* Bruch & Schimp.	Least Concern
	*Campylopusflexuosus* (Hedw.) Brid.	Least Concern
	*Campylopuspilifer* Brid.	Least Concern
	*Campylopuspyriformis* (Schultz) Brid.	Least Concern
	*Campylopusshawii* Wilson	Least Concern
	*Cyclodictyonlaetevirens* (Hook. & Taylor) Mitt.	Least Concern
	*Daltonialindigiana* Hampe	Endangered
	*Dicranumscottianum* Turner	Least Concern
	*Fissidensdubiu*s P.Beauv.	Least Concern
	*Fissidenstaxifolius* Hedw. subsp. taxifolius	Least Concern
	*Hylocomiumsplendens* (Hedw.) Schimp.	Least Concern
	*Hypnumcupressiforme* Hedw.	Least Concern
	*Kindbergiapraelonga* (Hedw.) Ochyra	Least Concern
	*Leucobryumglaucum* (Hedw.) Ångstr.	Least Concern
	*Leucobryumjuniperoideum* (Brid.) Müll.Hal.	Least Concern
	*Myuriumhochstetteri* (Schimp.) Kindb.	Least Concern
	*Polytrichumcommune* Hedw.	Least Concern
	*Polytrichumformosum* Hedw.	Least Concern
	*Ptychostomumcapillare* (Hedw.) Holyoak & N.Pedersen	Least Concern
	*Rhytidiadelphusloreus* (Hedw.) Warnst.	Least Concern
	*Sematophyllumsubstrumulosum* (Hampe) E.Britton	Least Concern
	*Sphagnumpalustre* L.	Least Concern
	*Sphagnumsubnitens* Russow & Warnst. subsp. subnitens	Least Concern
	*Thuidiumtamariscinum* (Hedw.) Schimp.	Least Concern
	*Tortellaflavovirens* (Bruch) Broth.	Least Concern
	*Trichostomumbrachydontium* Bruch	Least Concern
		
**b) Phylum: Marchantiophyta**		
Azorean endemic	*Bazzaniaazorica* H.Buch & Perss.	Endangered
	Leptoscyphusporphyriussubsp.azoricus (H.Buch & Perss.) Vanderp. & Heinrichs	Endangered
Macaronesian endemic	*Radulawichurae* Steph.	Near Threatened
	*Telaraneaazorica* (H.Buch & Perss.) Pócs	Endangered
European endemic	*Frullaniamicrophylla* (Gottsche) Pearson	Least Concern
	*Frullaniateneriffae* (F.Weber) Nees	Least Concern
	*Marchesiniamackaii* (Hook.) Gray	Least Concern
	*Porellacanariensis* (F.Weber) Underw.	Least Concern
	*Radulaaquilegia* (Hook.f. & Taylor) Gottsche, Lindenb. & Nees	Least Concern
	*Radulacarringtonii* J.B.Jack	Near Threatened
	*Radulaholtii* Spruce	Near Threatened
	*Saccogynaviticulosa* (L.) Dumort.	Least Concern
Native	Blepharostomatrichophyllum(L.)Dumort.subsp.trichophyllum	Least Concern
	*Calypogeiaarguta* Nees & Mont.	Least Concern
	Calypogeiafissa(L.)Raddisubsp.fissa	Least Concern
	Calypogeiamuelleriana(Schiffn.)Müll.Frib.subsp.muelleriana	Least Concern
	Cephaloziabicuspidata(L.)Dumort.subsp.bicuspidata	Least Concern
	*Cephaloziellabaumgartner*i Schiffn.	Least Concern
	*Cephalozielladivaricata* (Sm.) Schiffn.	Least Concern
	*Cephaloziellahampeana* (Nees) Schiffn. ex Loeske	Least Concern
	*Cephaloziellarubella* (Nees) Warnst.	Least Concern
	*Cololejeuneaazorica* V.Allorge & Jovet-Ast	Vulnerable
	*Cololejeuneamicroscopic*a (Taylor) Schiffn.	Least Concern
	*Cololejeuneasintenisii* (Steph.) Pócs	Endangered
	*Coluracalyptrifolia* (Hook.) Dumort.	Least Concern
	*Diplophyllumalbicans* (L.) Dumort.	Least Concern
	*Drepanolejeuneahamatifolia* (Hook.) Schiffn.	Least Concern
	*Frullaniaacicularis* Hentschel & von Konrat	Near Threatened
	Fuscocephaloziopsisconnivenssubsp.connivens (Dicks.) Vána & L.Söderstr.	Least Concern
	*Fuscocephaloziopsiscrassifolia* (Lindenb. & Gottsche) Vána & L.Söderstr.	Least Concern
	*Geocalyxgraveolens* (Schrad.) Nees	Near Threatened
	Harpalejeuneamolleri(Steph.)Grollesubsp.molleri	Least Concern
	*Herbertusazoricus* (Steph.) P.W.Richards	Endangered
	*Jubulahutchinsiae* (Hook.) Dumort. subsp. hutchinsiae	Least Concern
	*Kurziapauciflora* (Dicks.) Grolle	Least Concern
	Lejeuneaflavasubsp.moorei (Lindb.) R.M.Schust.	Near Threatened
	Lejeunealamacerina(Steph.)Schiffn.subsp.lamacerina	Least Concern
	*Lejeuneapatens* Lindb.	Least Concern
	Lepidoziacupressina(Sw.)Lindenb.subsp.cupressina	Least Concern
	*Lepidoziareptans* (L.) Dumort.	Least Concern
	*Lophocoleacoadunat*a (Sw.) Mont.	Least Concern
	*Lophocoleafragran*s (Moris & De Not.) Gottsche, Lindenb. & Nees subsp. fragrans	Least Concern
	Lophocoleaheterophylla(Schrad.)Dumort.subsp.heterophylla	Least Concern
	*Metzgeriafurcata* (L.) Corda	Least Concern
	*Microlejeuneaulicina* (Taylor) Steph.	Least Concern
	*Mniolomafuscum* (Lehm.) R.M.Schust.	Vulnerable
	Myriocoleopsisminutissima(Sm.)R.L.Zhu, Y.Yu & Pócssubsp.minutissima	Least Concern
	*Nowelliacurvifolia* (Dicks.) Mitt.	Least Concern
	Odontoschismadenudatum(Mart.)Dumort.subsp.denudatum	Least Concern
	*Odontoschismasphagni* (Dicks.) Dumort.	Least Concern
	*Pelliaepiphylla* (L.) Corda subsp. epiphylla	Least Concern
	*Plagiochilabifari*a (Sw.) Lindenb.	Least Concern
	*Plagiochilaexigua* (Taylor) Taylor	Least Concern
	*Plagiochilapunctata* (Taylor) Taylor	Least Concern
	*Pseudomarsupidiumdecipiens* (Hook.) Grolle	Least Concern
	*Riccardiachamedryfolia* (With.) Grolle	Least Concern
	*Scapaniagracilis* Lindb.	Least Concern
	*Telaraneaeuropaea* J.J.Engel & G.L.Merr.	Least Concern
